# Attitudes and practice patterns for maintaining relative dose intensity of chemotherapy in outpatient clinics: results of a Japanese web-based survey

**DOI:** 10.1186/s12885-015-1651-9

**Published:** 2015-10-05

**Authors:** Hitomi Sakai, Noriyuki Katsumata, Genmu Kadokura

**Affiliations:** Department of Medical Oncology, Nippon Medical School Musashikosugi Hospital, 1-396, Kosugi-machi, Nakahara-ku, Kawasaki City, Kanagawa 211-0063 Japan

## Abstract

**Background:**

This analysis was undertaken to evaluate the practice patterns of Japanese physicians regarding curative-intent chemotherapy, especially in outpatient settings, and to define factors negatively affecting the maintenance of relative dose intensity (RDI).

**Methods:**

We performed a web-based questionnaire survey of Japanese physicians involved in malignant lymphoma chemotherapy (Group ML) or in breast cancer chemotherapy (Group BC). The questionnaire inquired how they manage low-risk febrile neutropenia (FN) caused by initial chemotherapy for diffuse large B-cell lymphoma(DLBCL) or by adjuvant chemotherapy for breast cancer in an outpatient setting.

**Results:**

Valid responses were obtained from 185 physicians in Group ML and 160 in Group BC. In Group ML, 76 % (*n* = 141) of the physicians were board-certified hematologists, while 82 % (*n* = 131) of the physicians in Group BC were board-certified surgeons. A significantly higher proportion of physicians in Group ML responded that “dose reduction is not required for the subsequent course of chemotherapy after the first episode of FN” than in Group BC (ML versus BC; 77 % versus 31 %; *P* < 0.001). Significantly higher proportions of physicians in Group ML were more likely to prophylactically administer antibiotics or granulocyte-colony stimulating factor (G-CSF; ML versus BC; antibiotics: 36 % versus 26 %, *P* = 0.049; G-CSF: 25 % versus 16 %, *P* = 0.047). Eighty six percent (*n* = 159) of Group ML and 70 % (*n* = 112) of Group BC responded that “emergency outpatient unit is open at all hours”.

**Conclusions:**

Japanese physicians are more likely to administer reduced doses of chemotherapy to patients with breast cancer than to patients with malignant lymphoma. Supportive infrastructures should be improved to ensure the provision of adequate chemotherapy to all cancer patients.

## Background

Maintaining dose intensity is important for achieving the full benefits of chemotherapy in patients with potentially curable non-Hodgkin’s lymphoma and breast cancer. In 1990, Epelbaum et al. reported a strong association between the relative dose intensity (RDI) of a standard CHOP (cyclophosphamide, doxorubicin, vincristine, prednisone) regimen and 5-year survival among 95 patients with diffuse large-cell lymphoma (DLCL) [1]. The 5-year survival rate was 80 % in patients who received more than the median average RDI, whereas it was only 32 % in those who received less than the median average RDI (*P* < 0.001). Similarly, analysis of the RDIs of three doxorubicin-based regimens (including CHOP) in 115 patients with DLCL revealed that RDI of doxorubicin greater than 75 % was the most important predictor of survival [2]. A recently published retrospective analysis by Bosly et al. showed that survival of patients with diffuse large B-cell lymphoma (DLBCL) improved with an increasing average RDI (ARDI) of CHOP-21. Median survival was 7.08 years in those who received >90 % of the ARDI, significantly longer than in those who received ≤90 % of the ARDI (*P* = 0.002) [3]. In 1981, Bonadonna et al. reported a clear dose–response effect for CMF (cyclophosphamide, methotrexate, and 5-fluorouracil [5-FU]) chemotherapy in 449 women with breast cancer [4]. Their results showed that patients receiving ≥85 % of the planned CMF dose had a 5-year relapse-free survival (RFS) rate of 77 %, compared with 48 % in patients receiving <65 % of the planned dose. In 1995, 20-year follow-up data from the same group confirmed that RFS and overall survival (OS) were substantially better in patients who received ≥85 % of their planned dose than in those who received lower doses [5].

In 1998, Budman et al. reported the results of a randomized trial of adjuvant CAF (cyclophosphamide, doxorubicin, 5-FU) for stage II breast cancer patients. In total, 1,550 breast cancer patients were randomly assigned to one of three treatment arms: high-, moderate-, or low-dose intensity treatments [6]. The results revealed that the patients who received high- or moderate-dose intensity had significantly longer disease-free survival (*P* < 0.001) and OS (*P* = 0.004) than those who received low-dose intensity.

Recently, some study protocols specify that patients who have an initial episode of febrile neutropenia (FN) should additionally receive granulocyte-colony stimulating factor (G-CSF) or prophylactic antibiotics in subsequent cycles, and dose modification of chemotherapy is unnecessary [7–9]. If there is a second FN episode despite G-CSF or antibiotic support, the protocols recommend a reduction in chemotherapy dose.

However, studies of patients with aggressive non-Hodgkin’s lymphoma and early-stage breast cancer in the United States have reported that nearly half of such patients receive reduced dose-intensity chemotherapy [10, 11]. Additionally, how Japanese physicians manage outpatient chemotherapy and apply supportive measures to maintain RDI remains largely unknown. In Japan, chemotherapy for malignant lymphoma has been traditionally administered by hematologists, while chemotherapy for breast cancer is administered mainly by surgeons. This study was designed to clarify physicians’ attitudes and practice patterns with respect to curative-intent chemotherapy and to define factors that negatively affect RDI maintenance in Japan.

## Methods

We posted a questionnaire on a Japanese web site for physicians. Registration was required to access the questionnaire and those who completed the questionnaire could receive points from the web site as an incentive. The target respondents were physicians involved in the treatment of malignant lymphoma (Group ML) and those involved in the treatment of breast cancer (Group BC). Respondents in Group ML had to: 1) be a member of the Japanese Society of Hematology; 2) work at a hospital with more than 20 beds; 3) attend more than five patients with Non-Hodgkin’s lymphoma who receive chemotherapy; and 4) attend at least one patient who received R-CHOP (rituximab, cyclophosphamide, doxorubicin, vincristine, prednisone) in the past year. Respondents in Group BC had to: 1) be a member of the Japanese Breast Cancer Society; and 2) attend more than 15 patients who received neoadjuvant or adjuvant chemotherapy in the past year. The number of current members of the Japanese Society of Hematology is around 6,400, whereas number of current members of the Japanese Breast Cancer Society is around 9,800, 68 % of which are surgeons.

In the questionnaire, we described a patient who received first-line chemotherapy for DLBCL in Group ML and a patient who received adjuvant chemotherapy for early breast cancer in Group BC. In the clinical scenarios, the patients suffer from low risk FN with The Multinational Association for Supportive Care in Cancer (MASCC) scores ≥21 [12, 13] and in Talcott group 4 [14]. The questionnaire inquired about the management of FN and subsequent cycles of chemotherapy. The questions asked in the survey are listed in Table 1. This survey was administered in Japanese. The surveillance period was from November 30 through December 11, 2012.

**Table 1:** Questions asked in the survey

Q. How old are you?
1. ≤29
2. 30–34
3. 35–39
4. 40–44
5. 45–49
6. 50–54
7. 55–59
8. ≥60
Q. In what decade did you receive your medical license?
1. 2000s
2. 1990s
3. 1980s
4. 1970s
Q. Please select one of the following to indicate your area of specialty.
(For Group ML)
1. Board-certified internist
2. Board-certified hematologist
3. Board-certified oncologist
4. Not applicable
(For Group BC)
1. Board-certified surgeon
2. Board-certified breast surgeon
3. Board-certified oncologist
4. Board-certified internist
5. Not applicable
Q. Please select one of the following to indicate your place of employment.
1. Academic medical center
2. Cancer center or public hospital
3. Private hospital
4. Other
Diffuse large B-cell lymphoma (DLBCL)
A 68-year-old woman was given a diagnosis of DLBCL, Stage IV A. There were hepatic metastases, but no bone marrow infiltration. She had no clinically significant past medical history. The International Prognostic Index was high-intermediate risk. Performance status (PS) was 0. Lactate dehydrogenase (LDH) was 1,250 IU/L. She was scheduled to receive six cycles of R-CHOP (rituximab 375 mg/m^2^ on day 1 or day 2, cyclophosphamide 750 mg/m^2^ on day 1, doxorubicin 50 mg/m^2^ on day 1, vincristine 1.4 mg/m^2^ on day 1 [max 2 mg], prednisone 100 mg on days 1–5) given every 21 days.
Breast cancer
A 68-year-old postmenopausal woman was given a diagnosis of right breast cancer, cT2N0M0 stage II A. She had no clinically significant past medical history. PS was 0. Right total mastectomy was performed. Pathological findings were as follows: pT 2.0 cm, grade 3, ly-, v-, pN1 (3/20), ER(-), PgR(-), HER2(-). She was scheduled to receive four cycles of TC (docetaxel 75 mg/m^2^ on day 1, cyclophosphamide 600 mg/m^2^ on day 1) given every 21 days.
Q1. Would you manage low-risk febrile neutropenia in patients such as those describe above on an inpatient or outpatient basis?
1. Outpatient
2. Inpatient
Q2. (For those who chose outpatient management) Which of the following choices do you feel most closely describes the treatment you usually provide to this type of patient?
1. Oral antibiotics only
2. Oral antibiotics and G-CSF
3. Observation
4. Other
Q3. (For those who chose inpatient management) Which of the following choices do you feel most closely describes the treatment you usually provide to this type of patient?
1. Intravenous antibiotics
2. Intravenous antibiotics and G-CSF
3. Other
[Clinical Course]
On the tenth day of the first cycle, she presented with a fever of 39 °C. A systematic review was unrevealing. Dietary and fluid intake was sufficient.
Blood pressure, 135/80 mmHg
HEENT: She had a clear oropharynx.
Chest: No rales or wheezes were present.
Cardiac: Normal S1 and S2. There was no murmur.
Abdomen: Soft and flat. Bowel sounds were normal.
Laboratory data: WBC:1,200/mm^3^, ANC:400/mm^3^, Hb:11.4 g/dL, PLT:158,000, GOT:23 IU/L, Alb:3.6 g/dL, BUN:18.8 mg/dL, Cr:0.6 mg/dL, CRP:1.8 mg/dL
Q4. How do you modify the dose of subsequent courses of chemotherapy after febrile neutropenia? Please select one of the following options.
1. Dose reduction is not required
2. Dose reduction is required if febrile neutropenia was treated by intravenous antibiotics
3. Dose reduction is required at any rate
4. Other
Q5. How do you use antibiotics for the subsequent course of chemotherapy after febrile neutropenia? Please select one of the following options.
1. Antimicrobial prophylaxis deserves consideration
2. Antibiotics should be taken into account when the next episode of febrile neutropenia occurs
3. I typically do not administer antibiotics
4. Other
Q6. How do you use G-CSF for the subsequent course of chemotherapy after febrile neutropenia? Please select one of the following options.
1. G-CSF prophylaxis deserves consideration
2. G-CSF should be taken into account when neutropenia occurs
3. G-CSF should be taken into account when the next episode of febrile neutropenia occurs
4. I typically do not administer G-CSF
5. Other
Q7. Regarding systems for managing adverse effects of outpatient chemotherapy, please check all appropriate responses.
1. Emergency outpatient unit is open at all hours
2. Clinical laboratory is open at all hours
3. Diagnostic imaging unit is open at all hours
4. Hospital antibiogram is available
5. Health professionals provide patient and family education
6. Chemotherapy telephone helpline is available
7. Not applicable

All survey data were coded and analyzed with the use of standard EZR (Saitama Medical Center, Jichi Medical University), which is a graphical user interface for R (The R Foundation for Statistical Computing, version 2.13.0) [15]. More precisely, it is a modified version of R commander (version 1.6–3) that includes statistical functions that are frequently used in biostatistics. For comparisons of categorical variables, Fisher’s exact tests were used.

The execution of the survey followed the ethical principles outlined in the Declaration of Helsinki regarding human clinical research. The approval of the Ethics Committee of Nippon Medical School Musashikosugi Hospital was not required. This is because the regulation of the Ethics Committee of Nippon Medical School does not stipulate that a questionnaire survey for physicians requires ethical committee approval. Moreover, this is an anonymous questionnaire survey and we only use pseudonymized data.

## Results

Table 2 lists the participant characteristics. Valid responses were obtained from 185 respondents in Group ML and 160 in Group BC; there were no invalid responses. In Group ML, 76 % (*n* = 141) of the respondents were board-certified hematologists, and 10 % (*n* = 18) were board-certified oncologists. In Group BC, 82 % (*n* = 131) were board-certified surgeons and 36 % (*n* = 58) were board-certified breast surgeons. Overall, 11 % (*n* = 17) of the respondents in Group BC were board-certified oncologists. In Group ML, 32 % (*n* = 59) of the respondents were working at academic medical centers, 32 % (*n* = 59) at cancer centers or public hospitals, and 36 % (*n* = 67) at private hospitals. In Group BC, 21 % (*n* = 33) were working at academic medical centers, 29 % (*n* = 46) at cancer centers or public hospitals, and 43 % (*n* = 69) at private hospitals.

**Table 2:** Demographic characteristics of respondents

Characteristic	Group ML (*n* = 185)			Group BC (*n* = 160)		
		Number	%		Number	%
Age (years)	≤29	5	3	≤29	1	1
	30–34	22	12	30–34	12	8
	35–39	30	16	35–39	33	21
	40–44	37	20	40–44	31	19
	45–49	43	23	45–49	39	24
	50–54	28	15	50–54	23	14
	55–59	13	7	55–59	16	10
	≥60	7	4	≥60	5	3
Decade of medical license	2000s	44	24	2000s	33	21
	1990s	88	48	1990s	72	45
	1980s	46	25	1980s	46	29
	1970s	7	4	1970s	9	6
Specialty	Board-certified internist	142	77	Board-certified surgeon	131	82
	Board-certified hematologist	141	76	Board-certified breast surgeon	58	36
	Board-certified oncologist	18	10	Board-certified oncologist	17	11
	Not applicable	15	8	Board-certified internist	10	6
				Not applicable	8	5
Type of clinic/hospital	Academic medical center	59	32	Academic medical center	33	21
	Cancer center or public hospital	59	32	Cancer center or public hospital	46	29
	Private hospital	67	36	Private hospital	69	43
	Other	0	0	Other	12	8

Table 3 summarizes how the respondents manage low-risk FN. 50 % (*n* = 93) of the physicians in Group ML chose outpatient treatment for FN as compared with 65 % (*n* = 104) in Group BC (*P* = 0.006). Among the respondents who chose outpatient treatment, a higher proportion of physicians chose both oral antibiotics and G-CSF in Group ML than in Group BC (82 % versus 53 %, *P* < 0.001). However, intravenous antibiotics and G-CSF were preferred among physicians who chose inpatient treatment for FN.

**Table 3:** Management of low-risk febrile neutropenia

		Group ML		Group BC		
		(*n* = 185)		(*n* = 160)		
		Number	%	Number	%	*P*-value
Q. Inpatient versus outpatient management						
	Outpatient	93	50	104	65	*P* = 0.006
	Inpatient	92	50	56	35	*P* = 0.006
	Total	185	100	160	100	
Q. (For those who chose outpatient management) Treatment of FN						
	Oral antibiotics only	14	15	47	45	*P* < 0.001
	Oral antibiotics and G-CSF	76	82	55	53	*P* < 0.001
	Observation	0	0	2	2	*P* = 0.499
	Other	3	3	0	0	*P* = 0.103
	Total	93	100	104	100	
Q. (For who choose inpatient management) Treatment of FN						
	Intravenous antibiotics only	9	10	5	9	*P* = 1
	Intravenous antibiotics and G-CSF	83	90	51	91	*P* = 1
	Other	0	0	0	0	*P* = 1
	Total	92	100	56	100	

*Abbreviations*: *FN* febrile neutropenia

Table 4 summarizes how the respondents modify the dose of chemotherapy in patients who have FN and their attitudes toward the use of antibiotics and G-CSF for subsequent cycles of chemotherapy. In Group ML, 77 % (*n* = 143) of the physicians responded that “dose reduction is not required” compared with 31 % (*n* = 49) in Group BC (*P* < 0.001). In Group BC, approximately one third of the physicians responded that “dose reduction is required if FN was treated by intravenous antibiotics” and another third responded that “dose reduction is required at any rate”. Thirty-six percent (*n* = 67) of Group ML and 26 % (*n* = 42) of Group BC responded that “antimicrobial prophylaxis deserves consideration” (*P* = 0.049). Approximately half of the physicians in each group responded that “antibiotics are taken into account on the next episode of FN”. Twenty-five percent (*n* = 47) of Group ML and 16 % (*n* = 26) of Group BC responded that “G-CSF prophylaxis deserves consideration” (*P* = 0.047). Approximately half of the physicians in each group responded that “G-CSF is taken into account when neutropenia occurs”. About one third of Group BC responded that “G-CSF is taken into account when the next episode of FN occurs”.

**Table 4:** Management of subsequent cycles of chemotherapy after low-risk FN

		Group ML (*n* = 185)		Group BC (*n* = 160)		
		Number	%	Number	%	*P*-value
Q. Dose of chemotherapy						
	Dose reduction is not required	143	77	49	31	*P* < 0.001
	Dose reduction is required if febrile neutropenia was treated by intravenous antibiotics	18	10	56	35	*P* < 0.001
	Dose reduction is required at any rate	22	12	55	34	*P* < 0.001
	Other	2	1	0	0	*P* = 0.501
	Total	185	100	160	100	
Q. Antibiotics						
	Antimicrobial prophylaxis deserves consideration	67	36	42	26	*P* = 0.049
	Antibiotics are taken into account on the next episode of febrile neutropenia	91	49	95	59	*P* = 0.065
	I typically don’t administer antibiotics	27	15	23	14	*P* = 1
	Other	0	0	0	0	*P* = 1
	Total	185	100	160	100	
Q. G-CSF						
	G-CSF prophylaxis deserves consideration	47	25	26	16	*P* = 0.047
	G-CSF is taken into account when neutropenia occurs	114	62	75	47	*P* = 0.006
	G-CSF is taken into account on the next episode of febrile neutropenia	15	8	46	29	*P* < 0.001
	I typically don’t administer G-CSF	7	4	13	8	*P* = 0.107
	Other	2	1	0	0	*P* = 0.501
	Total	185	100	160	100	

Table 5 shows the details of the systems used to manage adverse effects of outpatient chemotherapy. For this analysis, physicians who work at clinics with less than 12 beds were not included in Group ML, but were included in Group BC. Eight percent (*n* = 12) of physicians in Group BC worked at clinics with less than 20 beds. Eighty-six percent (*n* = 159) of Group ML and 70 % (*n* = 112) of Group BC responded that the “emergency outpatient unit is open at all hours”. Sixty-nine percent (*n* = 128) of Group ML and 41 % (*n* = 66) of Group BC responded that the “clinical laboratory is open at all hours”. Moreover, 63 % (*n* = 117) of Group ML and 33 % (*n* = 52) of Group BC responded that the “diagnostic imaging unit is open at all hours”. Only 15 % (*n* = 27) of physicians in Group ML and 16 % (*n* = 26) of those in Group BC group responded that a “chemotherapy telephone helpline is available”.

**Table 5:** System for managing adverse effects during outpatient chemotherapy

		Group ML (*n* = 185)		Group BC (*n* = 160)		
		Number	%	Number	%	*P*-value
Q. Regarding the system for managing adverse effects of outpatient chemotherapy, please check all appropriate responses						
	Emergency outpatient unit is open at all hours	159	86	112	70	*P* < 0.001
	Clinical laboratory is open at all hours	128	69	66	41	*P* < 0.001
	Diagnostic imaging unit is open at all hours	117	63	52	33	*P* < 0.001
	Hospital antibiogram is available	105	57	40	25	*P* < 0.001
	Health professions provide patient and family education	81	44	52	33	*P* = 0.035
	Chemotherapy telephone helpline is available	27	15	26	16	*P* = 0.765
	Not applicable	0	0	11	7	*P* < 0.001

## Discussion

The most important finding of our study is that many Japanese physicians reduce the dose of chemotherapeutic agents after the first episode of low-risk FN in patients with potentially curable aggressive non-Hodgkin’s lymphoma or early-stage breast cancer. In the questionnaire, we presented the case of a patient who had FN during treatment for aggressive non-Hodgkin’s lymphoma or early-stage breast cancer in an outpatient setting (Table 1). She was clinically stable without significant medical comorbidity on presentation. Her MASCC score [12, 13] was 24, and she was classified as Talcott’s Group 4 [14], indicating low-risk FN. As for the subsequent course of chemotherapy, a higher proportion of physicians in Group BC responded that “dose reduction is required at any rate” or that “dose reduction is required if FN was treated by intravenous antibiotics” than in Group ML.

As mentioned in the introduction, there is well-established evidence supporting the clinical significance of RDI and its impact on survival in patients with aggressive non-Hodgkin’s lymphoma or early stage breast cancer [1–6]. This is why reducing the dose and delaying chemotherapy should be avoided. FN and severe prolonged neutropenia can lead to the decision to reduce chemotherapy dose and delay subsequent treatment cycles. In addition, the risk of fatal infection rises as the absolute neutrophil count falls below 500/mm^3^ and is higher in those with a prolonged neutropenia duration (>7 days) [16]. Therefore, management of afebrile and febrile neutropenia is significant. The Cochrane Haematological Malignancies Group published a review that compare the effectiveness of prophylactic administration of G-CSF or Granulocyte Macrophage Colony-Stimulating Factor (GM-CSF) with antibiotics in cancer patients receiving chemotherapy [17]. Two randomized controlled trials were eligible. This review showed non-significant results favoring antibiotics for preventing fever or hospitalization for FN compared with G-CSF. However, in one of the two trials, the chemotherapy dose intensity received by the antibiotic comparison group was much lower than in the GM-CSF group [18], which may explain the increased incidence of infections in the GM-CSF group. A non-randomized comparison within a randomized controlled trial (GEPARTRIO study) lead to a different outcome [19]. In breast cancer patients receiving TAC (docetaxel, doxorubicin and cyclophosphamide) pegfilgrastim alone or pegfilgrastim plus antibiotics provided suboptimal protection against FN and antibiotics alone was least effective.

Our results showed that that G-CSF and antibiotics are not commonly administered as prophylaxis against FN by Japanese physicians. G-CSF use for the management of established afebrile neutropenia was preferred in both groups. Guidelines recommend against the use of G-CSF in patients with afebrile neutropenia [20–23]. A randomized, double blind, placebo-controlled trial of G-CSF has been performed in afebrile outpatients with severe chemotherapy-induced neutropenia [24]: G-CSF shortened the duration of neutropenia, but did not decrease the hospitalization rate for FN, length of hospital stay, the number of days of antibiotic therapy, and the likelihood of having a positive culture.

Guidelines support the use of G-CSF in patients with FN who are at high risk for infection-associated complications [20–23]. A randomized, open-label, non-placebo-controlled trial has evaluated the effectiveness of adding G-CSF to antibiotic therapy in patients with solid tumors and chemotherapy-induced high-risk FN [25]. Adding G-CSF to antibiotic therapy was found to shorten the duration of neutropenia and reduce the duration of antibiotic therapy and hospitalization, but the treatment success rate, time to fever resolution, and mortality rate were similar in both treatment arms. Contrary to such evidence, many physicians use G-CSF with therapeutic intent.

In Japan, the majority of cancer care, including chemotherapy for solid tumors, has been historically performed by surgeons. Moreover, there is a shortage of medical oncologists in Japan. As of 2015, only 954 physicians have become Board-Certified Medical Oncologists of the Japanese Society of Medical Oncology (JSMO) [26]. Oncology education and training system in Japan needs much improvement. In addition, pegfilgrastim was not available in Japan until November 2014, and hospital visits on successive days were required. These factors may have a negative impact on outpatient management of chemotherapy and supportive care.

The Japanese Breast Cancer Society has developed Clinical Practice Guidelines for the systemic treatment of breast cancer [27]. These guidelines do not report how to use G-CSF or antibiotics as curative-intent chemotherapy. Including information about RDI and supportive measures into these guidelines may be an effective way to improve maintenance of dose-intensity.

About 50 % of Group ML and 35 % of Group BC chose to have the patient admitted to hospital for the treatment of FN. The American Society of Clinical Oncology (ASCO) clinical practice guidelines recommends outpatient management of low-risk FN as an option for carefully selected patients [28]. Based on the ASCO’s members’ expert opinion, “access to a telephone and transportation 24 h a day” is one of the requirements for outpatient treatment. However, our survey revealed that support systems for outpatient chemotherapy have not been adequately established in many hospitals and clinics in Japan.

Our study has several important limitations. First, the respondents may have been forgetful or may have responded without understanding the full context of the situation presented in the survey. In addition, eligible respondents were limited to physicians who had access to the website, potentially introducing self-selection bias. Despite these limitations, we believe that our study represents an important step in the improvement of cancer chemotherapy in Japan.

## Conclusions

In summary, our results suggest that supportive measures to deliver full dose-intensity chemotherapy are not widely used by Japanese physicians. Systems to support outpatient chemotherapy should thus be improved.

## Abbreviations

RDI: Relative dose intensity
ML: Malignant lymphoma
BC: Breast cancer
FN: Febrile neutropenia
DLBCL: Diffuse large B-cell lymphoma
G-CSF: Granulocyte-colony stimulating factor
DLCL: Diffuse large-cell lymphoma
ARDI: Average relative dose intensity
5-FU: 5-fluorouracil
RFS: Relapse-free survival
OS: Overall survival
MASCC: Multinational Association for Supportive Care in Cancer
GM-CSF: Granulocyte Macrophage Colony-Stimulating Factor
ASCO: American Society of Clinical Oncology
